# Choroidal and Choriocapillaris Changes after Photodynamic Therapy and Subthreshold Micropulse Laser Treatment for Central Serous Chorioretinopathy

**DOI:** 10.3390/medicina60101674

**Published:** 2024-10-12

**Authors:** Maria Ludovica Ruggeri, Marta Di Nicola, Marzia Passamonti, Carolina Lorenzi, Alberto Quarta, Rodolfo Mastropasqua, Lisa Toto

**Affiliations:** 1 Ophthalmology Clinic, Department of Medicine and Science of Ageing, “G. d’Annunzio” University Chieti-Pescara, 66100 Chieti, Italy; 2 Laboratory of Biostatistics, Department of Medical, Oral and Biotechnological Sciences, “G. d’Annunzio” University Chieti-Pescara, 66100 Chieti, Italy; 3 Department of Neuroscience, Imaging and Clinical Science, “G. d’Annunzio” University Chieti-Pescara, 66100 Chieti, Italy

**Keywords:** central serous chorioretinopathy, choroid, optical coherence tomography, optical coherence tomography angiography, Photodynamic Therapy, Subthreshold Micropulse Laser

## Abstract

*Background and Objectives*: The aim of the present study is to analyze choroidal and choriocapillaris structural and functional changes in eyes affected by Central serous chorioretinopathy after Photodynamic Therapy (PDT) and Subthreshold Micropulse laser (SML) treatment. *Materials and Methods*: Forty-two eyes of forty-two patients were analyzed in this observational study. Twenty-four patients underwent SML treatment, whereas eighteen patients were treated with PDT. Examinations were performed at baseline and after 3 months of treatment. Main outcome measures were: Best corrected visual acuity (BCVA), central macular thickness (CMT), central choroidal thickness (CCT), pigment epithelial detachment (PED) presence and maximum height (PEDMH), and choroidal vascularity index (CVI) measured by means of Spectralis HRA + OCT (Heidelberg Engineering, Heidelberg, Germany) Optical coherence tomography (OCT) and choriocapillaris flow voids (CCFV) measured on Optical Coherence Tomography Angiography (OCT-A) platform PLEX Elite 9000 device (Carl Zeiss Meditec Inc., Dublin, CA, USA). *Results*: Changes in BCVA were registered in both groups over time (*p* < 0.001). Structural changes in terms of reduced CMT and PED presence were noted in the two groups at follow-up (*p* < 0.001 and *p* = 0.001, respectively). Structural and functional choroidal changes were found in the two groups with reduced CCT and CVI over time (*p* = 0.004 and *p* = 0.007, respectively), with significant differences between the two groups for CVI parameter (*p* = 0.001). CCFV increased over time in the PDT group and decreased in the SML group. *Conclusions*: PDT and SML are effective approaches in CSC eyes and are able to improve structural and functional parameters over time. Choroidal and choriocapillaris parameters are promising biomarkers able to monitor disease course, showing greater impact of PDT on choroid-choriocapillaris complex over time.

## 1. Introduction

Central serous chorioretinopathy (CSC) is a chorioretinal disorder characterized by reduced visual acuity and metamorphopsia. The condition is associated with the presence of subretinal fluid (SRF) and a thicker choroid, thus showing it as part of the pachychoroid spectrum [1,2]. Although the central role of corticosteroids has been clearly identified in the pathogenesis of disease, the process is mainly attributable to choroidal hyperpermeability, which can extensively be assessed through Indocyanine Green Angiography (ICGA) study [3]. Consistent with these reports, Kuroda et al. found CSC eyes to be characterized by diffuse choroidal thickening with middle and large sized choroidal vessels, thus underlining the central role of the choroid in the pathogenesis of the disease [4]. To date, different treatments are available for active CSC, all reporting high efficacy and safety profile. In our previous study, we analyzed choroidal vascularity index (CVI) changes after oral eplerenone treatment in patients diagnosed with chronic CSC, thus demonstrating the ability of the mineral corticoid receptor antagonists in recovering choroidal morphology and supporting the key role of choroidal biomarkers in disease monitoring [5]. Consistent with our results, previous studies have found oral Eplerenone to be effective in CSC treatment, reporting restoration of choroidal and retinal morphology in affected eyes. Similarly, Photodynamic Therapy (PDT) is a well-known treatment that has shown overall positive results in the treatment of active CSC. It entails the use of verteporfin photosensitive dye, which is able to transform light into chemical energy and whose high RPE affinity allows choriocapillaris (CC) and vascular remodeling, causing decreased choroidal hypopermeability [6]. In addition, an increasing number of studies have been published on Subthreshold Micropulse laser (SML) safety and efficacy in CSC, highlighting its ability in inducing Retinal pigment epithelium (RPE) stimulation through short pulses of light without damaging adjacent neuroretina [7]. A recent study by Oribio-Quinto et al. has reported decreased Subfoveal Choroidal Thickness (SFCT) in CSC patients undergoing SML, which the authors determine to be attributable to the increased number of functional RPE cells [8]. Both PDT and SML appear to have a role in choroidal remodeling following treatment, although this has not been completely assessed. To fulfill this need, most recent devices, such as Optical Coherence Tomography Angiography (OCT-A), have allowed us to obtain a deeper knowledge of disease by detecting CC hypoperfusion in the chronic stages [9]. Consistent with these results, Burnasheva et al., in their artifact free study of CC in patients affected by CSC, analyzed CC perfusion decrease occurring in diseased eyes. Interestingly, in their paper, the CC hypoperfusion is recorded in the fellow eye as well, despite the presence of asymptomatic structural RPE changes or the acute form of disease being present [10]. All these data support the idea of CSC as part of the pachychoroid spectrum disease, thus acknowledging the occurrence of choroidal modifications in affected eyes, which have shown evidence of either choroidal hyperpermeability at ICG and/or choroidal thickening on Optical coherence tomography (OCT) [11,12]. In a recent study, Borrelli et al. analyzed choroidal changes in fellow eyes of patients affected by unilateral central serous chorioretinopathy, reporting the presence of inner and outer choroidal changes in both eyes of patients with unilateral disease. Interestingly, in their analysis, they found subtle RPE changes to be related to a greater CC hypoperfusion, evidencing the necessity of studying choroidal and CC modifications occurring in both eyes of patients affected by unilateral CSC [13]. Thus, the aim of the present study is to investigate choroidal and choriocapillaris changes in patients affected by unilateral CSC by comparing eyes undergoing PDT and SML treatment.

## 2. Materials and Methods

A total of 42 treatment naive eyes diagnosed for active central serous chorioretinopathy (CSC) were analyzed in our observational study. All patients were enrolled at the Ophthalmology Clinic of University “G. d’Annunzio”, Chieti- Pescara, Italy, from January 2023 to January 2024. The study complied with the tenets of the Declaration of Helsinki and specific informed consent was obtained from all patients. Inclusion criteria were: diagnosis of acute CSC in at least one eye confirmed by fundus examination with indirect ophthalmoscopy and multimodal imaging; idiopathic CSC with less than 1 month of symptoms duration; no previous local treatments (including laser treatment, photodynamic therapy, or intravitreal injections); any active disease at presentation other than CSC.

Exclusion criteria were: previous treatments; history of any other ocular diseases; autoimmune or other systemic diseases affecting the eye; ocular media opacities according to Lens Opacities Classification System III; patients aged less than 18 years old; local or general diseases other than CSC; previously reported CSC episodes. Twenty-four patients were treated with SML whereas eighteen patients were treated with PDT. All affected eyes were analyzed at baseline (T0) and 3 months after treatment (T1). All patients underwent complete ophthalmologic examination, including best corrected visual acuity (BCVA) expressed in logarithm of the minimum angle of resolution (logMAR), intraocular pressure (IOP) with Goldman applanation tonometry, slit lamp biomicroscopy, fundus examination with indirect ophthalmoscopy after 1% tropicamide instillation and fundus autofluorescence (FAF), fluorescein angiography (FA), indocyanine green angiography (ICGA), spectral domain optical coherence tomography (SD-OCT), and optical coherence tomography angiography (OCTA). FA, ICGA, FAF, and multicolor imaging were performed at baseline and they were found to be compatible with CSC diagnosis, whereas BCVA, anterior segment biomicroscopy, intraocular pressure, indirect fundus exam, SD-OCT, and OCTA were performed at baseline (T0) and then after 3 months of treatment (T1). Main outcome measures were BCVA; central macular thickness (CMT); central choroidal thickness (CCT); pigment epithelial detachment (PED) and maximum height (PEDMH); choroidal vascularity index (CVI); and choriocapillaris flow voids (CCFV).

### 2.1. SD-OCT Analysis

SD-OCT scans were performed using the Spectralis HRA + OCT (Heidelberg Engineering, Heidelberg, Germany). A 49 horizontal raster dense linear B-scans centered on the fovea, alongside horizontal and vertical B-scans with enhanced depth imaging (EDI) mode, were acquired. Images with poor signal strength (<25) were excluded and repeated. CMT was determined by measuring the thickness within the central 1 mm diameter circle of the ETDRS thickness map. CCT was measured vertically from the outer border of the RPE to the inner border of the sclera and measured using the inbuilt manual caliper on EDI OCT scans under the fovea on the horizontal macular line B-scan passing through the fovea. PED was defined as the separation between the RPE (Retinal Pigment Epithelium) and Bruch’s membrane and was manually measured at its maximum height (PED-MH) using the caliper tool. Measurements were taken from the scan revealing the most prominent lesion site within the central 6-mm-diameter circle of the ETDRS grid. CVI was calculated using a validated algorithm [14]. EDI-OCT horizontal and vertical single line scans centered on the fovea were exported and, once the choroid was manually identified, the limits of the ROI were set, whose area was representative of the total choroidal area (TCA). Images were binarized using Niblack’s auto local threshold; dark pixels were defined as the luminal choroidal area (LCA) while white pixels were defined as stromal choroidal area (SCA). CVI was obtained as the ratio between LCA and TCA. 

### 2.2. OCTa Analysis

OCTa analysis was performed using a validated described protocol [14]. Images were acquired with a swept-source OCT-A platform PLEX Elite 9000 device (Carl Zeiss Meditec Inc., Dublin, CA, USA). En face structural and OCTA CC images were extracted. Images were imported and analyzed in Image J software version 1.52 (National Institutes of Health, Bethesda, MD, USA; available at http://rsb.info.nih.gov/jj/index.html). Choriocapillaris flow analysis was performed by obtaining the final four binarized images (original CC image binarized with a radius of four pixels, original CC image binarized with a radius of eight pixels, compensated CC image binarized with a radius of four pixels, and compensated CC image binarized with a radius of eight pixels), which were processed to count and measure flow deficits [13]. 

### 2.3. SML Protocol

Navigated SML was performed with Navilas^®^ Laser System 577s Prime (Navilas^®^ 577s; OD-OS GmbH, near Berlin, Germany) as previously described [5]. A 577-nm yellow laser treatment was released and a system for navigated focal and peripheral laser treatments was used (OD-OS GmbH, Warthestr. 21 14513 Teltow, Germany). All patients underwent the standard protocol with 100 μm spot size and 100 ms duration with 5% duty cycle, although power was individualized in every patient after energy titration in a normal area of the retina outside the vascular arcade. The final laser treatment power was set at 30% of titrated energy and then delivered to the corresponding leakage areas on mid-phase FFA or mid-phase ICGA images that were imported to the laser device, superimposed, and aligned with the live image by means of an eye tracking system. 

### 2.4. PDT Protocol

All patients received 3 mg/m^2^ body surface area of verteporfin (Visudyne; Novartis AG, Basel, Switzerland) by infusion over a period of 10 min. After five minutes following the drug infusion, a PDT laser (Zeiss Visulas 690s PDT Laser System, Carl Zeiss Meditec, Inc., AG, Jena, Germany) with a 689-nm wavelength and power of 50 J/cm^2^ was used to deliver the treatment to the affected area for a duration of 83 s via a PDT laser lens. The identification of the pathological region was determined based on the largest linear measurement of choroidal hyperpermeability area visualized through ICGA. Patients were instructed to avoid bright lights and sunlight for 72 h after PDT.

### 2.5. Statistical Analysis

Statistical analyses were performed using R software environment for statistical computing and graphics (version 4.2; http://www.r-project.org/). Descriptive statistics were reported as median and interquartile range (first quartiles; third quartiles) for continuous variables, while categorical data were summarized as absolute frequency and percentage. The Shapiro–Wilk test was performed to verify normal distribution of data. A linear mixed model for repeated measurements was employed to assess the impact of group and time, as well as the interaction between these factors, on the primary outcome measures. Mixed model is a powerful method for analyzing data from longitudinal studies, in which there are multiple measurements on each subject. This method considers the correlation between repeated measurements on the same subject and enables explicit modeling of within-person and between-person variance in the outcome. Baseline and three months measures were regressed on the fixed-effect components, assuming an unstructured covariance matrix, using a linear mixed model for repeated measurements. A post-hoc power analysis respect to BCVA as primary outcome was conducted. Considering, in our mixed linear model for repeated measures, an effect size (f) of 0.25, our sample of 42 eyes would achieve 88% power. We set a significance level (α) at 0.05, correlation among the repeated measures (T0 and T1) at 0.5, and nonsphericity correction ε at 1. All tests were two-tailed, with a significance level set at *p* ≤ 0.05.

## 3. Results

**Figure 1:**
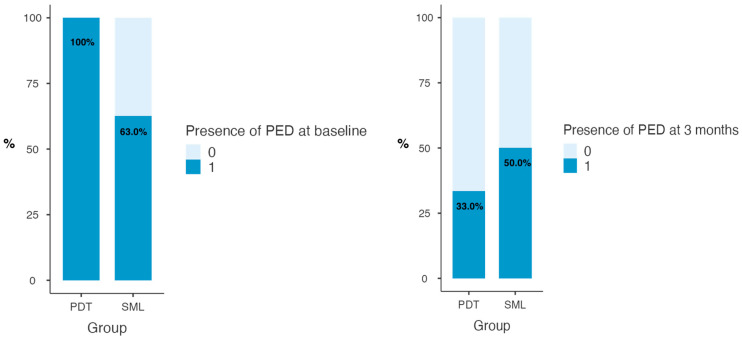
Percentage of presence of PED at baseline and at 3 months of treatment in two groups. McNemar test *p* < 0.001.

**Figure 2:**
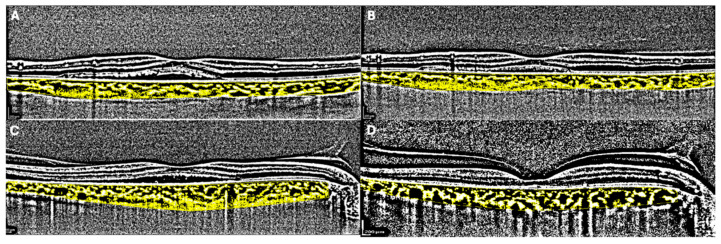
Choroidal changes in SML group before (**A**) and after treatment (**B**) measured by CVI. Changes in CVI before (**C**) and after (**D**) PDT are reported.

Forty-two eyes from forty-two patients suffering from CSC were enrolled in our observational study. Median age was 51.0 (50.0–55.5) years for the SML group and 60.0 (56.0–61.0) years for the PDT group (*p* = 0.138). Patients’ clinical characteristics are reported in Table 1. Overall median BCVA improved significantly in both groups over time (*p* < 0.001), with significant differences between the two groups (*p* = 0.033) in favor of PDT treatment. In the structural OCT analysis, CMT reduced in both groups (*p* < 0.001) without statistically significant differences between the two. PED presence decreased significantly over time (*p* = 0.001) while PED-MH did not show significant reduction (Figure 1). Choroidal parameters presented interesting trends over time. On OCT evaluation, CCT showed overall reduced parameters over time in both groups (*p* = 0.004), with greater clinical impact in the PDT group. Consistently, an overall significant CVI reduction was registered in both groups (*p* = 0.007), with greater reduction in the PDT group when compared to the SML group (*p* = 0.004) (Figure 2). Similarly, flow deficit analysis was revealed to be higher in the PDT group after treatment when compared to the SML group. 

## 4. Discussion

The study of choroid in CSC has always been of interest due to the physiopathological role covered in the disease. Different findings have been reported identifying choroidal filling delay, vascular dilation, and hyperpermeability to be characteristics of CSC, thus confirming choroidal circulatory alterations primary involvement in the pathogenesis of disease [15]. In fact, the choroidal hyperpermeability, which drives the event cascade clinically leading to reduced visual acuity and metamorphopsia, has been identified as possible target of treatment throughout the years. As a consequence, a wide range of effective therapies targeting the choroid have been developed, which to date are able to significantly improve the course of disease. The course, however, appears to be heterogeneous, ranging from self-limitans to relapsing remitting cases. Thus, the necessity of establishing well defined treatment able to control disease inducing regression appears to be evident. PDT and SML are both valuable treatments that are widely accepted worldwide. A recent study by Gao et al. has highlighted SML efficacy and safety by comparing it with oral spironolactone in chronic CSC, finding a lower recurrence rate and fewer adverse events in the SML group. In their study, better recovery of the choroidal structure was found in the spironolactone group [16]. An OCT study by Sawaguchi et al. noted half dose PDT efficacy in inducing and maintaining reduced choroidal thickness after 1 year of treatment [17]. Despite being different and not completely explored therapeutical mechanisms, both options have shown to be successful in treating CSC, with supporting research and clinical studies [18]. However, while PDT directly aims at modifying choroidal hyperpermeability through a direct choriocapillaris vessels occlusion, SML aims at stimulating RPE by regulating permeability factors and thus improving RPE pump function with decreased fluid over time. Consistent with these findings, in our study, both functional and structural parameters improved over time in the two groups, with better overall impact in favor of PDT. In fact, although BCVA showed significant improvements in both groups over time, PDT patients experienced a greater increase. Structural OCT parameters showed a similar trend, with CMT and PED decreasing over time in both groups, thus highlighting the effectiveness of the two treatments despite the presence of higher baseline PED values in the PDT group. As the choroidal vasculature is the physiopathological driver leading to disease, and based on the two different therapeutical mechanisms, it was our hypothesis that choroidal changes may be interpreted as a mirror of the disease dynamic over time, shedding light on the possibility of monitoring CSC through choroidal structural and functional study. Specifically, in our study, changes in choroidal parameters were noted over time, with reduced CCT and CVI in both groups, showing significant reduction in the PDT group when compared to the SML one. CCT is a validated parameter that has, overall, demonstrated reliability in modifying according to disease course in CSC patients. As recently reported by Koizumi et al., dilated large choroidal vessels manifest with thickened choroid in CSC eyes; however, according to their theory, the authors speculate the sclera to be responsible for inducing choroidal circulatory disturbances in CSC eyes, proposing development of minimally invasive treatments targeting the sclera in cases of refractory CSC [15]. This is consistent with Maruko et al., who recently speculated congenital scleral changes and focal choroidal thickening to affect the pathophysiology of CSC [19]. The relevance of CVI parameters has already been acknowledged in different studies, being a parameter able to represent both functional and structural choroidal impairment without being significantly influenced by systemic and local parameters. Xia et al. have recently remarked CVI reliability as biomarker in CSC, finding it to be increased in CSC eyes and fellow eyes of acute CSC cases. Moreover, the authors did not find significant differences in CVI in both the acute and chronic forms of disease [20]. Changes occurring in the choroid may reflect the course of disease and thus move differently depending on treatment. Being representative of both functional and structural choroidal aspects, we hypothesize the greater parameters reduction in PDT group rather than SML to be consequent to PDT mechanical efficacy in influencing choroidal vasculature, which is given by the therapeutical mechanism on which PDT relies on. Besides, it is worth highlighting that both treatments have shown effectiveness in reducing choroidal parameters, thus confirming both their efficacy in the disease and as possible biomarkers of disease over time. Nevertheless, we must acknowledge that by treating early CSC patients, part of the disease resolution may be attributable to the time effect rather than to the applied treatment, thus representing a limitation of our study when interpreting PDT and SML effectiveness, which have, overall to date, been widely accepted. In fact, the aim of the present analysis is to further elucidate PDT and SML therapeutical mechanism by studying their efficacy in modulating choroid and choriocapillaris through a study analysis aimed at investigating choroid and choriocapillaris changes occurring after treatment. Borrelli et al. reported the occurrence of a higher number of flow voids in the OCTa analysis in patients affected by CSC and fellow eyes. Their analysis has been further validated by the evidence of consistent results despite the different analysis method performed [13]. Interestingly, in our study, the number of flow deficits moves differently between the two groups over time, increasing in PDT patients when compared to SML. This may be derived from the PDT mechanism, which has further been confirmed by histopathological studies demonstrating choriocapillaris occlusion following PDT due to platelet aggregation and inflammatory substances that are consequent to oxidative materials generated by light exposure and verteporfirin [21]. The reliability of flow voids as efficient biomarkers in evaluating PDT effectiveness in CSC patients has already been investigated by Fernandez-Vigo et al., who reported increased flow signal voids immediately after PDT to be a good biomarker that is able to predict SFR resorption [22]. Consistent with their results, we hypothesize the higher number of flow deficits recorded after PDT treatment to be reflective of the physiopathological mechanism underlying PDT and thus enhancing the key role of choriocapillaris in CSC pathogenesis, follow up, and treatment. 

## 5. Conclusions

Our findings open up the possibility of identifying new choroidal biomarkers that may reflect the course of disease and thus be helpful in disease monitoring and treatment planning, confirming the central role of choroid in CSC and highlighting PDT effectiveness in modifying the choroidal physiopathological driver of disease.

## Figures and Tables

**Table 1:** Characteristics at baseline and 3 months in the two groups. Data are expressed as median and interquartile range (Q1–Q3).

Variable	Baseline		T1 (3 Months of Treatment)		*p*-Value		
	SML	PDT	SML	PDT	Time ^+^	Group ^#^	Interaction ^†^
BCVA (logMAR)	0.10 (0.10–0.20)	0.15 (0.02–0.20)	0.10 (0.0–0.10)	0.0 (0.0–0.0)	<0.001	0.033	0.245
CMT (µm)	257.0 (233.7–387.5)	251.0 (186.2–318.7)	206.5 (138.0–213.5)	181.0 (151.0–214.0)	<0.001	0.069	0.237
CCT (µm)	353.0 (279.7–453.7)	369.0 (354.7–453.0)	331.5 (296.2–396.0)	288.5 (229.5–412.0)	0.004	0.823	0.012
PED max height (µm)	88.0 (52.5–128.7)	145.0 (100.2–165.7)	87.5 (71.7–114.7)	91.5 (65.7–117.2)	0.115	0.853	0.101
CVI	0.68 (0.67–0.69)	0.67 (0.66–0.70)	0.69 (0.67–0.69)	0.64 (0.62–0.65)	0.007	0.001	0.004
CCFV	113.9 (96.7–179.0)	76.7 (56.2–93.0)	70.8 (62.0–95.1)	170.4 (101.9–238.1)	0.038	0.040	0.037

SML: Subthreshold Micropulse Laser; PDT: Photodynamic Therapy; BCVA: Best Corrected Visual Acuity; CMT: Central Macular Thickness; CCT: Central Choroidal Thickness; PED-MH: Pigment Epithelium detachment maximum height; CVI: Choroidal Vascularity Index; CCFV: Choriocapillaris flow voids. ^+^ time, for each variable, the differences have been tested between the median of each time point of the two groups. ^#^ group, for each variable, the differences have been tested between the median of SML group at two times (baseline and T1) and the median of the PDT group at two times (baseline and T1). ^†^ Probability that the variation of parameter during the follow-up is different in one distinct group (interaction time × group).

## Data Availability

Data are unavailable due to privacy or ethical restrictions.
